# The global establishment of a highly-fluoroquinolone resistant *Salmonella enterica* serotype Kentucky ST198 strain

**DOI:** 10.3389/fmicb.2013.00395

**Published:** 2013-12-18

**Authors:** Simon Le Hello, Amany Bekhit, Sophie A. Granier, Himel Barua, Janine Beutlich, Magdalena Zając, Sebastian Münch, Vitali Sintchenko, Brahim Bouchrif, Kayode Fashae, Jean-Louis Pinsard, Lucile Sontag, Laetitia Fabre, Martine Garnier, Véronique Guibert, Peter Howard, Rene S. Hendriksen, Jens P. Christensen, Paritosh K. Biswas, Axel Cloeckaert, Wolfgang Rabsch, Dariusz Wasyl, Benoit Doublet, François-Xavier Weill

**Affiliations:** ^1^Unité des Bactéries Pathogènes Entériques, Centre National de Référence des Escherichia coli, Shigella et Salmonella, Institut PasteurParis, France; ^2^Biochemistry Department, Faculty of Pharmacy, Minia UniversityMinia, Egypt; ^3^Unité Caractérisation et épidémiologie Bactérienne, Laboratoire de Sécurité des Aliments, Université Paris-EstAnses, Maisons-Alfort, France; ^4^Department of Microbiology, Faculty of Veterinary Medicine, Chittagong Veterinary and Animal Sciences UniversityChittagong, Bangladesh; ^5^Unit Antimicrobial Resistance and Resistance Determinants, National Reference Laboratory for Antimicrobial Resistance, Department Biological Safety, Federal Institute for Risk AssessmentBerlin, Germany; ^6^Department of Microbiology, National Veterinary Research InstitutePuławy, Poland; ^7^National Reference Centre for Salmonellae and other Bacterial Enteric Pathogens, Robert Koch InstituteWernigerode, Germany; ^8^Centre for Infectious Diseases and Microbiology – Public Health, University of Sydney, ICPMRSydney, NSW, Australia; ^9^Institut Pasteur du Maroc, Sécurité alimentaire et EnvironnementCasablanca, Maroc; ^10^Department of Microbiology, University of IbadanIbadan, Nigeria; ^11^Bio Chêne Vert, FINALABChâteaubourg, France; ^12^WHO Collaborating Center for Antimicrobial Resistance in Foodborne Pathogens and European Union Reference Laboratory for Antimicrobial Resistance, Technical University of DenmarkKgs. Lyngby, Denmark; ^13^Department of Veterinary Disease Biology, Faculty of Health and Medical Sciences, University of CopenhagenFrederiksberg, Copenhagen, Denmark; ^14^Institut national de la recherche agronomique, UMR1282 Infectiologie et Santé publiqueNouzilly, France; ^15^Université François Rabelais de Tours, UMR1282 Infectiologie et Santé publiqueTours, France

**Keywords:** *S.* Kentucky, ST198, SGI1, QRDR, MDR *Salmonella* dissemination, poultry

## Abstract

While the spread of *Salmonella enterica* serotype Kentucky resistant to ciprofloxacin across Africa and the Middle-East has been described recently, the presence of this strain in humans, food, various animal species (livestock, pets, and wildlife) and in environment is suspected in other countries of different continents. Here, we report results of an in-depth molecular epidemiological study on a global human and non-human collection of *S.* Kentucky (*n* = 70). We performed *Xba*I-pulsed field gel electrophoresis and multilocus sequence typing, assessed mutations in the quinolone resistance-determining regions, detected β-lactam resistance mechanisms, and screened the presence of the *Salmonella* genomic island 1 (SGI1). In this study, we highlight the rapid and extensive worldwide dissemination of the ciprofloxacin-resistant *S.* Kentucky ST198-X1-SGI1 strain since the mid-2000s in an increasingly large number of contaminated sources, including the environment. This strain has accumulated an increasing number of chromosomal and plasmid resistance determinants and has been identified in the Indian subcontinent, Southeast Asia and Europe since 2010. The second substitution at position 87 in GyrA (replacing the amino acid Asp) appeared helpful for epidemiological studies to track the origin of contamination. This global study provides evidence leading to the conclusion that high-level resistance to ciprofloxacin in *S.* Kentucky is a simple microbiological trait that facilitates the identification of the epidemic clone of interest, ST198-X1-SGI1. Taking this into account is essential in order to detect and monitor it easily and to take rapid measures in livestock to ensure control of this infection.

## Introduction

Despite the substantial progress made in preventing foodborne diseases, new pathogens have emerged, some of which have spread worldwide decade after decade (Tauxe, 1997). These pathogens include strains of multi-drug resistant (MDR) *Salmonella* (Arlet et al., 2006; Walsh and Fanning, 2008). Their treatment in both animals and humans has become more difficult and the number of reports of foodborne infections and outbreaks of MDR *Salmonella* has increased (Angulo et al., 2000; Mølbak, 2005). The global spread of an MDR *Salmonella enterica* serotype Typhimurium phage type DT104 in animals and humans since the 1990s (Threlfall, 2000; Mather et al., 2013) is a good example. While the spread of DT104 may have been facilitated by the use of antimicrobials, the national and international trade of infected animals is thought to have played a major role in its spread across borders (Ribot et al., 2002; Weill et al., 2006a).

More recently, an emerging *S.* Kentucky strain has been described (Weill et al., 2006b; Le Hello et al., 2011) and belonged to the ST198-X1 subtype. It has accumulated various chromosomal resistance determinants since the mid-1990s with the integration of the *Salmonella* genomic island 1 (SGI1), a 43-kilobase genomic island initially described in DT104 (Boyd et al., 2001), encoding resistance to multiple antimicrobials including amoxicillin, gentamicin, and sulfonamides (Doublet et al., 2008), followed by cumulative mutations in the *gyrA* and *parC* genes, leading to resistance to nalidixic acid and then to ciprofloxacin in 2002 (*S.* Kentucky CIP-R). This population was mostly detected in Egypt before 2005, but has now rapidly spread throughout Africa and the Middle East (Le Hello et al., 2011). Another matter of concern is the expanding livestock reservoir of this *S.* Kentucky CIP-R strain. It was initially identified in autochthonous poultry but was then found in various animals and foods (contaminated spices in France and the United States of America (US), turkey flocks in Germany and Poland, wild animals, etc) (Le Hello et al., 2011; Beutlich et al., 2012; Münch et al., 2012; Wasyl and Hoszowski, 2012). Several isolates give rise to considerable concern, as they have become producers of various carbapenemases and/or cephamycinase and/or extended spectrum β-lactamases (ESBL) (Le Hello et al., 2013).

Since these studies, several reports have mentioned *S.* Kentucky CIP-R isolates that have been identified from different sources (animals, food, the environment and humans) and geographic locations, in particular in several new countries in the Indian sub-continent and Southeast Asia. The purpose of this study was to examine whether the *S.* Kentucky strains isolated around the world from different ecosystems belong to this expanding ST198-X1-SGI1 strain.

## Materials and methods

### Collection of strains

Following the publication of the first studies on *S*. Kentucky ST198-X1-SGI1 CIP-R surveillance (Weill et al., 2006b; Le Hello et al., 2011, 2013), public health agencies in several different regions have notified the same isolation of *S*. Kentucky CIP-R in their animal flocks, in the environment, in food and in humans (traveler-related or domestic). We studied 70 *S*. Kentucky strains from the following participating countries: 41 strains collected from the French National Reference Center for *Salmonella* (FNRC-Salm), 11 from the French Food Safety Agency *Salmonella* network (ANSES Laboratory for Food Safety, Maisons-Alfort, France), six from Denmark (five from the Veterinary Clinical Microbiology Section, Department of Veterinary Disease Biology, University of Copenhagen and one from the Technical University of Denmark, DTU), five from Germany (three from the National Reference Laboratory for *Salmonella*, Federal Institute for Risk Assessment, BfR, and two from the German National Reference Center for Salmonellae and other Enterics, RKI), three from the NSW Enteric Reference Laboratory, ICPMR, Westmead Hospital, Sydney (Australia), two from the National Veterinary Research Institute (Poland) and one from the Institut Pasteur of Morocco, and the reference strain 98K (Edwards, 1938) from the WHO Collaborating Center for Reference and Research on *Salmonella*, Paris, France.

The global collection, presented in Table 1, gathers *S*. Kentucky isolates from around the world (four continents, 28 countries) covering a long time-span (including preantibiotic era isolates, from 1937 to 2013), various livestock species (swine, turkey, layer, and broiler poultry farms), humans, food (soya bean, meat, seafood, and spices), wild and domestic animals (reptiles, horses, camels, birds, dogs) and the environment (compost and rivers).

**Table 1:** **Characteristics of the *S. enterica* serotype Kentucky isolates that constitute the representative Kentucky ST198 collection in this study**.

**Numbering**	**Year of isolation**	**Country of contamination**	**Sources**	**AST^a^**	***bla* genes^b^**	**Asp87 substitution in GyrA**	**CIP MIC mg/L**	**PFGE-type**	**MLST**	**SGI1^c^**	**References^d^**
**HISTORICAL STRAINS (FROM 1937 TO 1999)**											
98K	1937	USA	Chicken	Susceptible	–	No	0.008	X4	198	Absence	Edwards, 1938
1–61	1961	Senegal	Human	Susceptible	–	No	0.016	X1d	198	Absence	Le Hello et al., 2011
1–66	1966	Vietnam	Human	R2	TEM	No	0.016	X18	198	Absence	id
1–68	1968	Senegal	Human	SSu	–	No	0.016	X1b	727	Absence	id
2–75	1975	Senegal	Human	Susceptible	–	No	0.023	X1b	198	Absence	id
5–76	1976	France	Soya bean	Susceptible	–	No	0.008	X4	198	Absence	id
19–85	1985	Egypt	meat	SSpSuCTe	–	No	0.016	X2b	198	J6	Le Hello et al., 2011
93–6429	1993	Indonesia	Human	Su	–	No	0.016	X2c	198	J4	Le Hello et al., 2012
96–11313	1996	Egypt	Human	SSpGSuTe	–	No	0.023	X1a	198	Ks	Le Hello et al., 2011
97–6819	1997	Egypt	Human	ASSpSuTe	TEM	No	0.008	X1a	198	Ks	id
97–11473	1997	Egypt	Human	R1-Nal	TEM	No^1^	0.125	X1k	198	Ks	id
99–2998	1999	Egypt	Human	HR1-Nal	CMY-2	Asn	0.125	X1t	198	Absence	id
**RECENT STRAINS (SINCE 2000)**											
**HUMAN**											
00–1059	2000	Egypt	Human	R2-Nal	TEM	No^1^	0.125	X1a	198	P1	id
01–2100	2001	Egypt	Human	R1-Nal	TEM	No^1^	0.125	X1a	198	K1	id
02–9866	2002	Egypt	Human	R1-NalCip	TEM	Asn^2^	8	X1a	198	Ks	id
04–4567	2004	Egypt	Human	R1-KTmpCNalCip	TEM	Gly^2^	4	X1g	198	K1	id
05–1016	2005	Kenya	Human	R3	–	Tyr^2^	4	X1a	198	Q2	id
05–4680	2005	Sudan	Human	SSpGSuTmpNalCip	–	Gly^2^	4	X1l	198	K4	id
07–1511	2007	Morocco	Human	R2-NalCip	TEM	Asn^2^	16	X1a	198	Ps	id
07–7991	2007	Tunisia	Human	R2-NalCip	TEM	Asn^2^	12	X1b	198	+	id
08–4705	2008	Iran	Human	R3	–	Asn^2^	12	X1a	198	+	id
08–5707	2008	Tanzania	Human	R1-NalCip	TEM	Tyr^2^	16	X1c	198	+	id
09–8391	2009	Morocco	Human	HR2-NalCip	CMY-2	Asn^2^	32	X1e	198	+	Le Hello et al., 2013
09–9322	2009	Egypt	Human	HR3-NalCipAzi	TEM-1 + CMY-2	Gly^2^	12	X1w	198	+	id
2010/00305	2010	Egypt	Human	KCTmpNalCip	–	Gly^2^	12	X1w	198	+	id
2010/00720	2010	Turkey	Human	HR4-NalCip	TEM-1 + CTX-M-1	Asn^2^	16	X1b	198	+	id
2010/01922	2010	Morocco	Human	HR5-NalCip	TEM-1 + VIM-2	Gly^2^	12	X1m	198	+	id
2010/05456	2010	Algeria	Human	HR6-NalCipAzi	CTX-M-15	Asn^2^	12	X1a	198	Qs	id
2010/07071	2010	Cote d'Ivoire	Human	R1-NalCip	TEM	Tyr^2^	12		198	+	This study
2010/07297	2010	Unknown	Human	ASul	PSE-1	No	0.016		198	+	This study
2010/07503	2010	India	Human	R1-NalCip	TEM	Tyr^2^	8	X1b	198	Ks	This study
**RECENT STRAINS (SINCE 2000) HUMAN**											
2010/08553	2010	Senegal	Human	R1-NalCip	TEM-1	Asn^2^	12		198	+	This study
2010/09778	2010	Libya	Human	R1-NalCip	TEM	Asn^2^	24		1680	+	This study
2011/00664	2011	Egypt	Human	R2-NalCipAzi	OXA-48	Gly^2^	8	X1w	198	+	Le Hello et al., 2013
2011/01683	2011	India	Human	R1-NalCip	TEM	Tyr^2^	>32		198	+	This study
2011/01801	2011	Mali	Human	HR2	CMY-2	No	0.016	X3	1679	Absence	This study
2011/06348	2011	India	Human	R1-NalCip	TEM	Tyr^2^	8		198	+	This study
201111973	2011	Cameroon	Human	R3	-	Gly^2^	12		198	Qs	This study
80-11-227-5139	2011	India	Human	R1-NalCip	TEM	Tyr^2^	6		198	+	This study
80-11-252-4482	2011	Iraq	Human	ASGSulCTeNalCip	TEM	Gly^2^	8		198	+	This study
80-11-309-2385	2011	Cambodia	Human	ASulTeNalCip	TEM	Asn^2^	8		198	+	This study
201203105	2012	Indonesia	Human	R1-NalCip	TEM	Asn^2^	32	X1a	198	Ks	This study
201205363	2012	Kuwait	Human	ASSpGTeNalCip	TEM	Gly^2^	16	X1l	198	Ks	This study
201207374	2012	Vietnam	Human	ASulTeNalCip	TEM	Asn^2^	32	X1c	198	Ks	This study
2013/01062	2013	Algeria	Human	HR7-NalCipAzi	TEM-1 + OXA-48	Asn^2^	8	X1b	198	Ks	This study
**NON HUMAN**											
BfR 05-04625	2005	Ethiopia	Swine	R3	–	Gly^2^	8	X1a	198	+	This study
07AF4403	2006	Ethiopia	Chicken	R1-NalCip	TEM	Gly^2^	12	X1a	198	Ks	Le Hello et al., 2011
Em 06-02339	2006	United Arab Emirates	Camel	R1-KNalCip	TEM	Gly^2^	8		198	+	Münch et al., 2012
Em 07-04654	2007	United Arab Emirates	Houbara	ASSpKTSulTeNalCip	TEM	Gly^2^	12		198	+	id
08-KS6	2008	Nigeria	Chicken	R1-NalCip	TEM	Gly^2^	12	X1a	198	Ks	Le Hello et al., 2011
08-015	2008	Morocco	Seafood	R1-TNalCip	Nd	Asn^2^	16	X1d	198	Ks	id
09-8745	2009	Togo	Chicken	R1-NalCip	TEM	Gly^2^	12		198	Ks	This study
K-50	2009	Bangladesh	Layer poultry farms	R1-NalCip	TEM	Tyr^2^	8	X1e	198	+	Barua et al., 2012
K-26	2009	Bangladesh	Layer poultry farms	R1-NalCip	TEM	Tyr^2^	16	X1e	198	Ks	id
BfR 10-02164	2010	Germany	Turkey meat	R1-NalCip	TEM	Tyr^2^	8	X1b	198	Ks	Beutlich et al., 2012
BfR 10-02979	2010	Germany	Reptile organs	R1-NalCip	TEM	Tyr^2^	12	X1n	198	Ks	id
1090/10	2010	Poland	Turkey meat	R2-NalCip	TEM	Tyr^2^	16	X1b	198	+	Zając et al., 2013
10CEB962	2010	France	Compost	R2-NalCip	TEM	Asn^2^	12		198	+	This study
10CEB8465	2010	Côte d'Ivoire	Poultry meat	R1-NalCip	TEM	Tyr^2^	8		198	+	This study
10CEB8748	2010	France	Horse placenta	R1-TmpNalCip	TEM	Asn^2^	16		198	+	This study
10CEB8766	2010	France	river	SSpGSulTeNalCip	–	Asn^2^	12		198	+	This study
**NON HUMAN**											
B-81	2010	Bangladesh	Broiler poultry farms	R1-NalCip	TEM	Tyr^2^	8	X1a	198	+	Barua et al., 2013
B-11	2010	Bangladesh	Broiler poultry farms	R1-TTmpNalCip	TEM	Tyr^2^	8	X1e	198	+	id
K-78	2010	Bangladesh	Layer poultry farms	R1-NalCip	TEM	Tyr^2^	8	X1i	198	+	Barua et al., 2012
2189/11	2011	Poland	Reptile	R2-NalCip	TEM	Gly^2^	16	X1a	198	+	Zając et al., 2013
11CEB3342	2011	France	Spice	R2-NalCip	TEM-1	Asn^2^	12		198	+	This study
11CEB4816	2011	France	Marinated turkey meat	R1-NalCip	TEM-1	Tyr^2^	12		198	+	This study
12CEB716	2012	France	Dog	R2-NalCip	TEM	Asn^2^	8		198	+	This study
12CEB4452	2012	France	Turkey farms	R1-NalCip	TEM-1	Asn^2^	16	X1f	198	Ks	Guillon et al., 2013
13CEB2160	2013	Poland	Turkey meat	R1-NalCip	TEM	Tyr^2^	8	X1x	198	Ks	This study

a R1: resistance to amoxicillin, A; streptomycin, S; spectinomycin, Sp; gentamicin, G; sulfamethoxazole, Su; and tetracycline, Te.

R2: resistance to A.

R3: no resistance associated with NalCip.

ceftriaxone, Cro; ceftazidime, Caz; cefoxitin, Fox; trimethoprim, Tmp; chloramphenicol, C; azithromycin, Azi; imipenem, Imp; kanamycin, K; tobramycin, T; netilmicin, N; amikacin, A; isepamicin, I.

HR1: ACroCazFoxSSuTmpCTe.

HR2: ACroCazFox.

HR3: ACroCazFoxSSpKTNCSuTmp.

HR4: ACroSSpGSuTe.

HR5: ACroCazFoxImpSSpKTNGAISuTe.

HR6: ACroCazSSpKTGNAISulTmp.

HR7: AImpSSpGSuTmpTe.

b Nd, not done; –, any bla gene found.

1 associated with the gyrA Ser83Phe substitution.

2 associated with the gyrA Ser83Phe substitution and parC Ser80Ile substitution.

c +, positive for SGI1; Js, Ks, Ps, and Qs, variants of SGI1.

d id, idem, same reference as above.

### Microbiological investigations

Serotyping was performed on the basis of the White-Kauffmann-Le Minor scheme (Grimont and Weill, 2007). We carried out antimicrobial susceptibility testing (AST) on all *S.* Kentucky isolates with the disk diffusion method, for a panel of 32 antimicrobial drugs (Bio-Rad, Marnes-La-Coquette, France). Using Etests (bioMérieux, Marcy l'′Etoile, France), we determined the minimum inhibitory concentration (MIC) of ciprofloxacin, as previously described (Le Hello et al., 2013). The results were then interpreted using the breakpoints of the Antibiogram Committee of the French Society for Microbiology (CA-SFM) which implements the EUCAST breakpoints. Resistance to ciprofloxacin is defined as having an MIC of more than 1 mg/L and being susceptible at an MIC of 0.5 mg/L or less, irrespective of isolate source (i.e., intestinal or extraintestinal).

For molecular typing, we performed multilocus sequence typing (MLST) on all *S.* Kentucky isolates and PulseNet standard pulsed-field gel electrophoresis (PFGE) of *Xba*I-digested chromosomal DNA on a selection of isolates (*n* = 50) (Le Hello et al., 2011; Achtman et al., 2012).

To determine the resistance mechanisms, we assessed the presence of β-lactam resistance genes (*bla*_*TEM*_, *bla*_*SHV*_, *bla*_*PSE*_, *bla*_*OXA*−1_ group, *bla*_*CMY*_, *bla*_*CTX*−*M*_, *bla*_*OXA*−48_, *bla*_*VIM*_, *bla*_*NDM*_, and *bla*_*KPC*_), plasmid-mediated quinolone resistance genes (known PMQR genes, *qnr*A, *qnr*B, *qnr*S, *qnr*D, *aac(6)′-Ib-cr*, *qep*A and the recent *oqx*A and *oqx*B), macrolide resistance genes (*mph*A) and *Salmonella* genomic island1 (SGI1) by PCR, as described previously (Le Hello et al., 2013; Li et al., 2013). Variants SGI1-K, P, and Q and J were differentiated in some strains (*n* = 28) by selected primers, as described in Table 2 and also described previously (Doublet et al., 2008; Le Hello et al., 2012).

**Table 2:** **PCR set (SGI1- Ks, -Ps, and -Qs)**.

**PCR No**.	**Primer**	**Nucleotide sequence (5'-3')**	**Tm(°C)**	**Size (bp)**	**Remark**
13	sulTER	AAGGATTTCCTGACCCTG	59	2040	+ for SGI1-Ks
	RvTn501	ATGGATGTGGTGGCTGAAGG	67.2		
A	FwS026	CGGTTTTGAATAAGAAGGCA	61.5	Variable	+ for SGI1-Ps
	OT4	CCAATGCTTAATCAGTGAGG	59.7		
23	OT3	ATGAGTATTCAACATTTCCG	56.5	2192	+ for SGI1-Ps and – for SGI1-Qs
	RvS044c	GTATTGTCGTCGGGATGATT	61.2		
C	FwS026	CGGTTTTGAATAAGAAGGCA	61.5	Variable (~1500–3000)	+ for SGI1-Qs
	RvS044c	GTATTGTCGTCGGGATGATT	61.2		

The quinolone resistance-determining region (QRDR) of *gyr*A, *gyr*B, *par*C, and *par*E (encoding subunits of the DNA gyrase and the topoisomerase IV) was sequenced in all *S*. Kentucky strains as described previously (Le Hello et al., 2013). The nucleotide and deduced amino acid sequences were analyzed and compared with sequences available from the National Center for Biotechnology Information.

## Results

The Table 1 presents the characteristics of serotype Kentucky isolates and summarizes the results of antimicrobial resistance and genomic typing.

### Molecular typing

**Figure 1:**
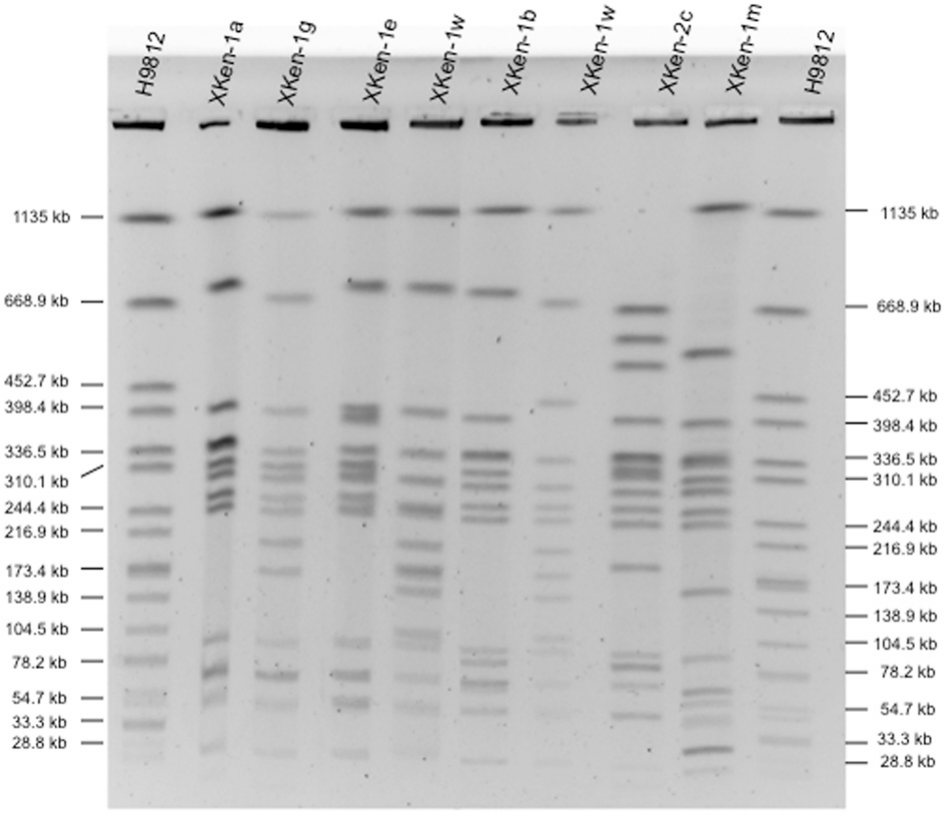
**Representatives of *Xba*I-PFGE profiles obtained among *S. enterica* serotype Kentucky isolates from humans and nonhumans during the period 1937–2013**.

All but one of the 70 *S*. Kentucky isolates belonged to the e-burst group 56: ST198 and two single locus variants (SLV), ST727 and ST1680. The remaining isolate belonged to a new ST, ST1679, sharing only three loci out of seven (this isolate was susceptible to quinolones and belonged to an X3 PFGE type). We distinguished diverse PFGE types among the ST198 and SLV isolates. The major one, X1 and its many variants (X1a–X1x), representing almost 90% of the ST198 isolates (*n* = 44), has been associated with strains isolated over the last 50 years, whereas X2 (*n* = 2/*N* = 50) has been linked to isolates from Asia isolated before the 2000s and several other patterns for older strains isolated before the 1980s (X4 for the reference strain 98K and strain 5–76 isolated from soya bean, and X18 for a Vietnamese strain, isolated in 1937, 1976, and 1966, respectively). Most common representative PFGE subtypes are shown in Figure 1.

The SGI1-K variants were firstly identified in Kentucky ST198 isolates from Egypt in 1996. Then, the SGI1-Ks and its derivative variants -Ps or -Qs were present in all the ST198 (or its SLV) isolates. As shown here, all the SGI1 variants were independently distributed between periods, countries and sources (Table 1).

### Antimicrobial susceptibility testing and resistance genes

Among the *S.* Kentucky isolates studied, increased resistance to fluoroquinolones has been observed since the isolation of the first ciprofloxacin-resistant isolate in 2002. As shown in Table 1 ciprofloxacin MICs seem to increase over time, from 0.008 mg/L to 0.125 mg/L during the 1990s, 4 mg/L to 12 mg/L between 2002 and 2006 and since 2007 with the emergence of highly ciprofloxacin resistant strains (16 to >32 mg/L). Apart from quinolone resistance, additional resistance was observed in some *S.* Kentucky CIP-R isolates (Table 1). The most prevalent drug resistance patterns were R1 (*n* = 25, 46%), which included resistance to amoxicillin, streptomycin, spectinomycin, gentamicin, sulfamethoxazole, and tetracycline; R2 (*n* = 8, 15%), which included resistance to amoxicillin; and R3 (*n* = 4, 7%), which included resistance only to nalidixic acid and ciprofloxacin. Before 2002, these resistance patterns were also described with decreased susceptibility to ciprofloxacin (CIP-DS; MIC, 0.125 mg/L). These patterns and other less frequent ones were all associated with the presence of SGI1-Ks (for R1) and -Ps (for R2), both carrying the *bla*_*TEM*_ gene, and -Qs (for R3 which do not carry any drug resistance genes).

More recently, additional resistance to third generation cephalosporins (C3G) and/or carbapenems was observed in CIP-R *S.* Kentucky X1-ST198-SGI1 isolates. These isolates contained the cephamycinase *bla*_*CMY*−2_ (*n* = 3), the ESBLs *bla*_*CTX*−*M*−1_ (*n* = 1) and *bla*_*CTX*−*M*−15_ (*n* = 1), and the carbapenemases *bla*_*VIM*−2_ (*n* = 1) and *bla*_*OXA*−48_ (*n* = 2). High-level resistance to azithromycin (32 mg/L to 128 mg/L) was found in four of them which carried the phosphotransferase *mph*A gene known to inactivate macrolide antimicrobial drugs.

In addition to the ST198-X1 SGI1-Ks, -Ps, and -Qs isolates, we observed other drug-resistant *S.* Kentucky ST198 populations in our collection. Initially, we identified ST198-X2, which carried SGI-Js and was isolated in Asia before the 2000s (see Le Hello et al., 2012); secondly, we identified here for the first time a putative SGI1-B variant carrying *S.* Kentucky ST198 isolate (no. 2010/07297) which contains a *bla*_*PSE*−1_ gene found occasionally in other serotypes such as Typhimurium DT104 and Paratyphi B d-tartrate fermenting (Boyd et al., 2002; Weill et al., 2005). Lastly, an SGI1 free isolate susceptible to nalidixic acid and producing the CMY-2 cephamycinase was acquired in Mali in 2011 (no. 2011/01801).

### Temporal and geographic distribution of *s*. kentucky isolates with QRDR mutations

**Figure 2:**
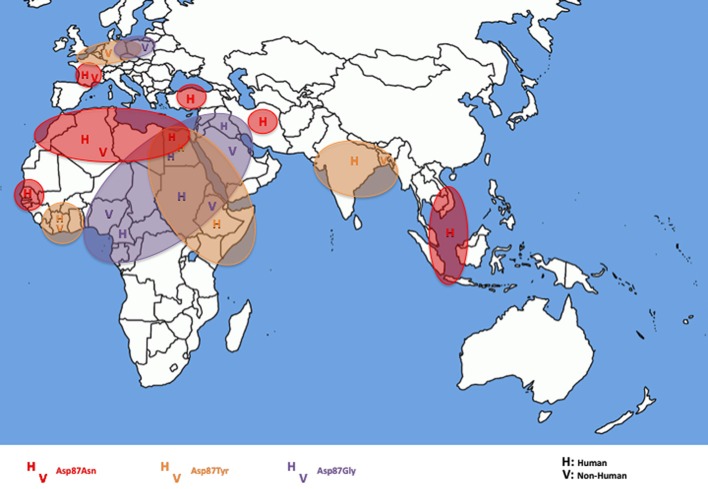
**Geographical distribution in the eastern hemisphere of the different substitutions at codon 87 of GyrA to asparagine (Asn), tyrosine (Tyr), or glycine (Gly) in *S. enterica* serotype Kentucky resistant to ciprofloxacin of the present collection**. H, Human isolates; V, nonhuman isolates.

As shown in Table 1, ciprofloxacin resistance in all the 54 CIP-R *S*. Kentucky isolates was related to GyrA and ParC substitutions (Table 1). All contained double substitutions in GyrA (at codons Ser83 and Asp87) and a single ParC substitution (Ser80 encoding an isoleucine residue). None of the isolates contained GyrB or ParE modifications. In GyrA, all the isolates contained phenylalanine at codon Ser83, whereas mutations in codon Asp87 resulted in different substitutions to asparagine (Asn), tyrosine (Tyr), or glycine (Gly) residues depending on the geographic origin of the isolates (Figure 2). All the Egyptian isolates presented three possible mutations in codon Asp87 (*n* = 6), whereas the isolates from North Africa (Morocco, Algeria, Tunisia, Libya) had a modification that resulted in Asp87Asn (*n* = 7). An exception was the VIM-2 producing Kentucky 2010/01922 isolate from Morocco which had Asp87Tyr. Those from the Middle East (Iraq, United Arab Emirates, Kuwait, Turkey, and Iran) presented both Asp87Gly and Asp87Asn, apart from two nonhuman Ethiopian isolates and one Sudanese human isolate in 2005 that had an Asp87Gly substitution; those from East Africa, India and Bangladesh had an Asp87Tyr modification (*n* = 12). Apart from the two strains from the Ivory Coast which presented Asp87Tyr, those from West Africa presented Asp87Gly (*n* = 3). All three isolates from Southeast Asia (Cambodia, Indonesia and Vietnam) had an Asp87Asn amino acid change.

Isolates from food, animal feed and the environment isolated in Europe presented an intermediate situation with isolates mostly having an Asp87Tyr residue in Germany and Poland and an Asp87Asn residue in France.

No PMQR genes, such as *qnr, aac(6′)-Ib-cr, qepA* and *oqxAB* were detected in *S*. Kentucky CIP-R isolates of this study.

### Nonhuman sources of the *s*. kentucky ST198-x1-SGI1 strain

The nonhuman isolates of *S*. Kentucky ST198-X1-SGI1 have mainly been found in poultry farms (chicken, layer and turkey, *n* = 14) since its first description in a chicken from Ethiopia in 2006 (Le Hello et al., 2011). This strain has further been described in poultry flocks in East, West and North Africa, the Indian sub-continent and Europe. It has also been described in food products (seafood, meat, or spice), domestic animals (dog, horse, camel, or pet reptiles) and wild animals (houbara or reptiles) and various environments (river or compost) (see Table 1).

## Discussion

By gathering and studying this global collection, we confirmed that one strain has disseminated throughout the developing countries in both human and nonhuman sources. Strain ST198-X1 displays high-level resistance to ciprofloxacin and harbors SGI1-Ks or its derivative variants -Ps or -Qs. The high-diversity of antibiotic resistance patterns could be related to genetic rearrangements mediated by various insertion sequences (in particular IS26) and transposons in SGI1-Ks, -Ps, and -Qs (Doublet et al., 2008). In parallel, contrary to the relatively few PFGE patterns associated with *S*. Typhimurium DT104, *S*. Kentucky ST198 displayed high-diversity in X1 subtypes, suggesting frequent events of genomic rearrangements present in SGI1 or the acquisition/loss of various plasmids. Several other ST198 strains belonging to other PFGE subtypes and/or containing different antibiotic resistance mechanisms have been isolated sporadically or have no longer been isolated since the 2000s (Le Hello et al., 2012). The odyssey of the *S*. Kentucky ST198-X1 isolate has lasted since it was found in Egypt in the mid-1990s. It has since then accumulated various chromosomal resistance determinants, with the integration of SGI1 (encoding resistance to multiple antimicrobial drugs), followed by cumulative mutations in the *gyr*A and *par*C genes, leading to resistance to nalidixic acid, and then to ciprofloxacin in 2002. Since 2002, this strain has spread rapidly throughout Africa and the Middle East (Le Hello et al., 2011) and, in 2009, it was identified in India in travelers and in Bangladesh in poultry flocks (Barua et al., 2012, 2013). Furthermore, the first description of SGI1 variant K was identified in a *Salmonella* serotype Kentucky strain isolated in 2001 from spices imported into Australia from India (Levings et al., 2007). Since 2011, a pattern of propagation across Asia is also suggested by the recent recovery of ciprofloxacin-resistant *S*. Kentucky isolates from stool samples of patients in France and Australia with a history of recent travel to Vietnam, Cambodia and Indonesia. This global collection also makes it possible to confirm the recent increase of *S*. Kentucky ST198-X1-SGI1 CIP-R clinical strains that have acquired additional genes, making them resistant to extended spectrum cephalosporins and/or carbapenems, in particular in the Mediterranean basin (Collard et al., 2007; Le Hello et al., 2013). High-level resistance to azithromycin due to the acquisition of the *mph*A gene among these isolates is of concern as this antimicrobial agent is presented as a good alternative treatment for severe *Salmonella* infections (Hill and Beeching, 2010) and it completes the variety of enteric bacteria already described producing this phosphotransferase (Boumghar-Bourtchai et al., 2008). Last but not least, as shown in this study, ciprofloxacin MIC has increased decade after decade in *S*. Kentucky isolates. The recent increase in MIC is not due to additional mutations in QRDR, nor to the presence of PMQR. However, the increase could be due to the overexpression of an efflux system such as AcrAB-TolC (Baucheron et al., 2013).

Another matter of concern is the expanding livestock reservoir of this *S*. Kentucky ST198-X1-SGI1 CIP-R strain, initially identified in African autochthonous poultry but subsequently found in various animals and food (Le Hello et al., 2011; Barua et al., 2012, 2013; Beutlich et al., 2012; Münch et al., 2012; Wasyl and Hoszowski, 2012). Poultry flocks have contributed to the global dissemination of this clone in developing countries since 2005. Hence this strain was isolated in Ethiopia in 2006 (chicken), Nigeria (chicken), and Morocco (turkey) in 2008 (Le Hello et al., 2011), in Togo (chicken) in 2009, and from layers in 2009, from broilers in 2010 in Bangladesh (Barua et al., 2012, 2013), and in developed countries since 2010 (Poland, Wasyl and Hoszowski, 2012; Germany, Beutlich et al., 2012; and France, Guillon et al., 2013). Interestingly, the *S*. Kentucky ST198-X1-SGI1 CIP-R identified in Europe was exclusively associated with turkeys. An investigation following the epidemics in Poland (Wasyl and Hoszowski, 2012) has established that at least one of the infected flocks was hatched from eggs imported from the Middle East. The diversity of nonhuman sources described in this study is another reason of concern. In particular, the description of *S*. Kentucky ST198-X1-SGI1 CIP-R in the environment (rivers and compost) and animals such as reptiles, indicates its potential long-term presence, suggesting its capacity to produce biofilm, as was previously identified in Tunisian isolates (Turki et al., 2012). This intrinsic characteristic could possibly facilitate secondary contaminations and persistence into a novel host/source but this remains to be investigated.

Regarding its recent establishment in Bangladesh (18% of 500 farms were *S*. Kentucky positive during the period 2009–2010), we do not have any information on how *S*. Kentucky ST198-X1-SGI1 CIP-R was introduced in poultry, although locally produced fish meal used as a protein source has been associated with the presence of *Salmonella* in flocks (Barua et al., 2012, 2013). Small poultry farmers purchase this raw ingredient from the local market to produce low-cost feed by mixing it with other ingredients. Meat and bone meal imported from different countries are also used for preparing poultry and fish feed as a source of protein. Regarding broiler chicks, they are supplied from commercial breeding farms. Further investigations are necessary to obtain information on locally produced poultry feed. The microbiological characteristics of Kentucky ST198-X1-SGI1 CIP-R isolated from poultry farms in Bangladesh indicated that the amino acid substitution in codon 87 of GyrA was exclusively a tyrosine residue like that found in Kentucky for India, East Africa, Egypt and in some Middle East countries. We do not know whether humans play any role in the introduction of this strain in various flocks. Regarding this, it is noteworthy that several million emigrants from Bangladesh work in the Middle East. The human role in the contamination of livestock by *S*. Kentucky ST198-X1-SGI1CIP-R has been pinpointed recently in local turkey flocks in France. The epidemiological investigation performed highlighted the introduction of this strain following the return of farmers from Morocco, a country where this strain is endemic, and suffering from diarrhea (Guillon et al., 2013). This hypothesis may be further reinforced as this isolate (12CEB4452) presented GyrA Asp87Asn substitution which is clearly associated with the North African *S*. Kentucky ST198-X1-SGI1 CIP-R, while isolate 13CEB2160, found in turkey meat sold in France but imported from Poland, presented tyrosine substitution, a characteristic described more frequently in Polish turkey flocks.

In the era of globalized food supply, livestock, and international human travel, the ciprofloxacin-resistant *S.* Kentucky ST198-X1-SGI1 strain is not restricted to one country, rendering measures to subject livestock to controls more difficult. National and international health, food, and agricultural authorities should include it among the strains targeted in national programs to control *Salmonella* spp in poultry. Based on different studies, including this one, the *S.* Kentucky ST198-X1-SGI1 epidemic clone of interest can nowadays easily be identified by simple serotyping (Kentucky) and by testing its susceptibility *in vitro* to ciprofloxacin (disk diffusion or MIC). It is essential to consider this strain at both national and international level, in order to take preventive measures as soon as possible to limit its worldwide propagation. Like this, we suggest including this emerging *Salmonella* in the European Union's list of target serotypes for mandatory monitoring (EU Commission regulations No.200/2012 of 8 March 2012).

## Author contributions

Simon Le Hello and François-Xavier Weill conceived and designed the experiments, analyzed the data, and wrote the report. Amany Bekhit, Lucile Sontag, Laetitia Fabre, Martine Garnier, and Véronique Guibert performed the experiments. The other Kentucky working group authors participated in the continuous monitoring of Kentucky strains, sent materials, participated in the discussion and reviewed the manuscript.

### Conflict of interest statement

The authors declare that the research was conducted in the absence of any commercial or financial relationships that could be construed as a potential conflict of interest.
